# Case Report: ALK-Positive Histiocytosis With *KIF5B-ALK* Fusion in Cerebrum-Disseminated Lesions in a Child

**DOI:** 10.3389/fonc.2022.858939

**Published:** 2022-03-10

**Authors:** Yi Guo, Hai-bo Qu, Gang Ning, Feng-lin Jia, Hong Liu, Xin-mao Ma, Yi Liao

**Affiliations:** ^1^ Department of Radiology, West China Second University Hospital, Sichuan University, Chengdu, China; ^2^ Key Laboratory of Birth Defects and Related Diseases of Women and Children (Sichuan University), Ministry of Education, Chengdu, China; ^3^ Department of Integrated Traditional and Western Medicine, Center of Hemorrhoid and Fistula, West China Hospital of Sichuan University, Chengdu, China

**Keywords:** ALK-positive histiocytosis, child, MRI, CT, brain, lung

## Abstract

**Background:**

Anaplastic lymphoma kinase (ALK)-positive histiocytosis is a rare type of histiocytosis that could affect multiple systems in children and adults. 10 cases of ALK-positive histiocytosis invading the central nervous system (CNS) have been reported. Herein, we report a case of ALK-positive histiocytosis invading the central nervous system and lungs and the details of follow-up of tumor dynamic changes during treatment.

**Case Presentation:**

An 18-month-old boy was underweight and had slow growth of almost 3 months duration. The child could not stand and walk independently, and his language and intelligence development occurred later than those of his peers. Cranial magnetic resonance imaging revealed a giant suprasellar lesion with isosignal, measuring approximately 5.1× 3.6× 4.0 cm on T1-weighted imaging, with an obvious mass effect. Nodular, slightly low-signal shadows were also observed in the left temporal pole and left hippocampus, measuring approximately 1.0 cm × 0.7 cm× 0.5 cm and 0.9 cm× 0.8 cm × 0.5 cm on T1-weighted, respectively. The child underwent partial resection of the suprasellar lesion, and a diagnosis of ALK-positive histiocytosis was made histologically. Subsequently, the patient received chemotherapy (CHOP regimen) and anti-ALK therapy (crizotinib). The lesions were gradually shrinking without dissemination and the changes of intracranial and lung lesions were monitored with imaging during therapy. Unfortunately, the child died 8 months after the first surgery because of worsening intracranial infection.

**Conclusion:**

ALK-positive histiocytosis may involve the central nervous system and disseminate intracranially. ALK-positive histiocytosis should be considered for the differential diagnosis of suprasellar lesions.

## Introduction

Anaplastic lymphoma kinase (ALK)-positive histiocytosis, which harbors a chromosomal translocation involving ALK at chromosome 2p23 (1), is a rare entity that can affect multiple systems, including the liver, spleen, digestive tract, bone marrow, skin, breast, and central nervous system (CNS) (2–5). ALK-positive histiocytosis can occur in both children and adults, while pediatric cases are more likely to have a disseminated disease that primarily affects the liver, spleen, or bone marrow (3). In adolescent patients, several symptoms can occur, including pallor, hepatosplenomegaly, thrombocytopenia, and anemia. When invading the CNS, ALK-positive histiocytosis can cause headache, vomiting, and seizures. According to a literature review of PubMed using keyword of ALK-positive histiocytosis, 10 cases of ALK-positive histiocytosis invading the CNS have been reported, and lesions located in the frontal lobe are more commonly reported (1, 3–7). Once the diagnosis of ALK-positive histiocytosis has been established, chemotherapy and anti-ALK therapy are generally applied. However, our review of the literature did not identify reports of imaging changes during treatment. Here, we report a rare case of intracranial disseminated ALK-positive histiocytosis in a child, mainly located in the suprasellar area, and discuss in detail the radiographic morphological changes occurring during chemotherapy and anti-ALK therapy.

## Case Description

An 18-month-old boy was underweight and had slow growth lasting for almost 3 months. On admission, the child’s height and weight were 0.74 m and 8.0 kg, respectively (body mass index = 14.6 kg/m^2^). No weight or height was gained despite increased feeding. The child could not stand and walk independently, and his language and intelligence development occurred later than do those of his peers. The boy was born in a natural birth and lived in Chengdu, Sichuan, China. He did not have any family history of congenital, allergic, or systemic diseases.

## Diagnosis, Intervention, and Outcome

### Clinical Examination

Alpha-fetoprotein (AFP) and beta-human chorionic gonadotropin (β-hCG) levels were negative. Other examinations were unremarkable.

### Imaging of Magnetic Resonance Imaging (MRI) and Computed Tomography (CT)

**Figure 1:**
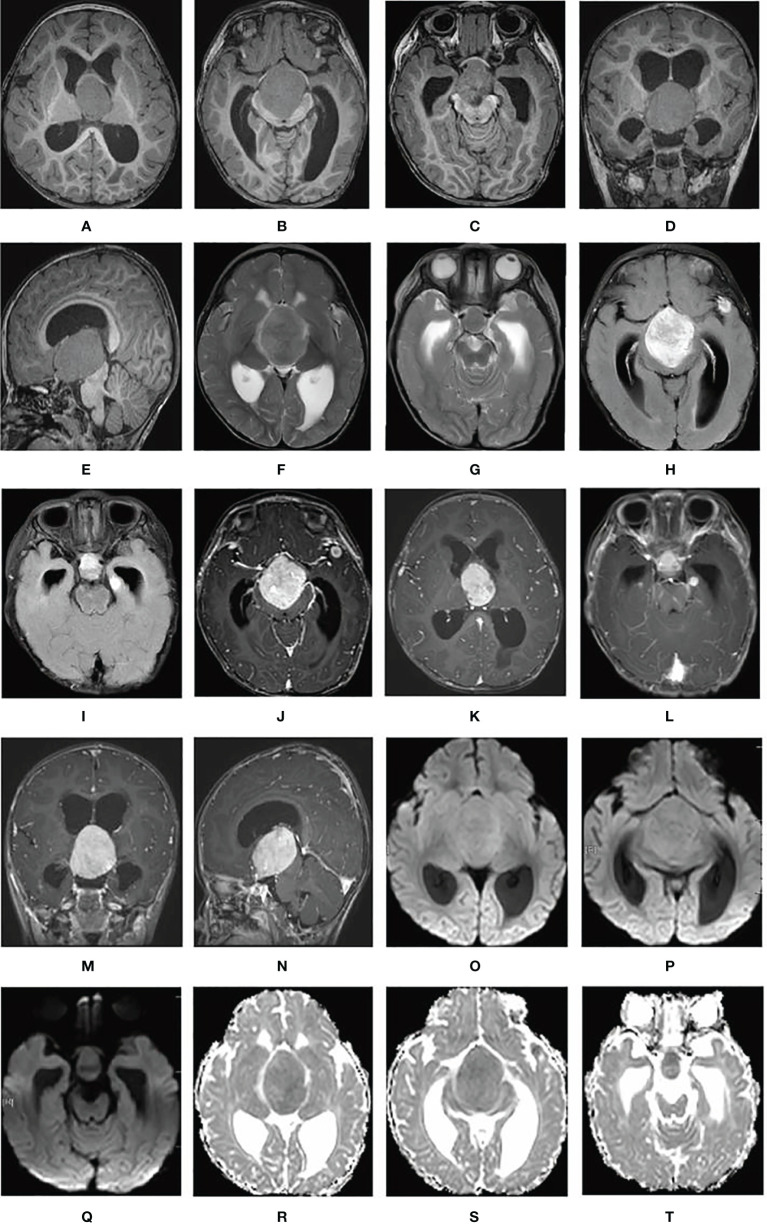
Cerebral lesions are seen on MRI. **(A–E)** T1-weighted MRI; **(F, G)** T2-weighted MRI; **(H, I)** fluid-attenuated inversion recovery MRI; **(J–N)** contrast-enhanced T1-weighted MRI; **(O–Q)** diffusion-weighted imaging; and **(R–T)** apparent diffusion coefficient.

T1-weighted cranial MRI (PHILIPS INGENIA 3.0T) revealed a giant suprasellar lesion with isosignal protruding toward the third ventricle and measuring approximately 5.1cm × 3.6 cm× 4.0 cm, with an obvious mass effect. The structures adjacent to the lesion, including the pituitary stalk, optic chiasm, and midbrain were significantly compressed. Bilateral lateral ventricular dilatation with significant hydrocephalus was also present. Two nodular, slightly low-signal shadows were observed in the left temporal pole and the left hippocampus, respectively, measuring approximately 1.0cm × 0.7cm × 0.5 cm and 0.9cm × 0.8cm × 0.5 cm, respectively (**Figures 1A–E**). All these lesions showed slightly high signal intensity on T2-weighted images and significantly high signal intensity on fluid-attenuated inversion recovery images (aka FLAIR) (**Figures 1F–I**). The supra-sellar nidus showed mild peripheral edema on T2-weighted MRI. The supra-sellar nidus also showed significant heterogeneous and compressed Willis rings on contrast-enhanced T1-weighted MRI. The left-temporal-pole lesion showed significant circumferential enhancement with low signal centrally, while the left hippocampal lesion showed significant enhancement on contrast-enhanced T1-weighted MRI (**Figures 1J–N**). All these lesions showed slightly higher signal on diffusion-weighted imaging and had a diminished apparent diffusion coefficient (**Figures 1O–T**).

Chest CT (Neusoft, NeuViz128) showed multiple nodules in the lungs (8–9 lesions in the left lung and 9–10 lesions in the right). The largest was located in the anterior segment of the upper lobe of the right lung and measured approximately 1.0 cm× 0.9 cm (**Figures 3A**–**F**). There were no enlarged lymph nodes in the hilum or mediastinum after enhancement. Abdominal CT showed an enlarged liver and a slightly enlarged spleen, but no abnormal density or foci of enhancement were observed.

**Figure 2:**
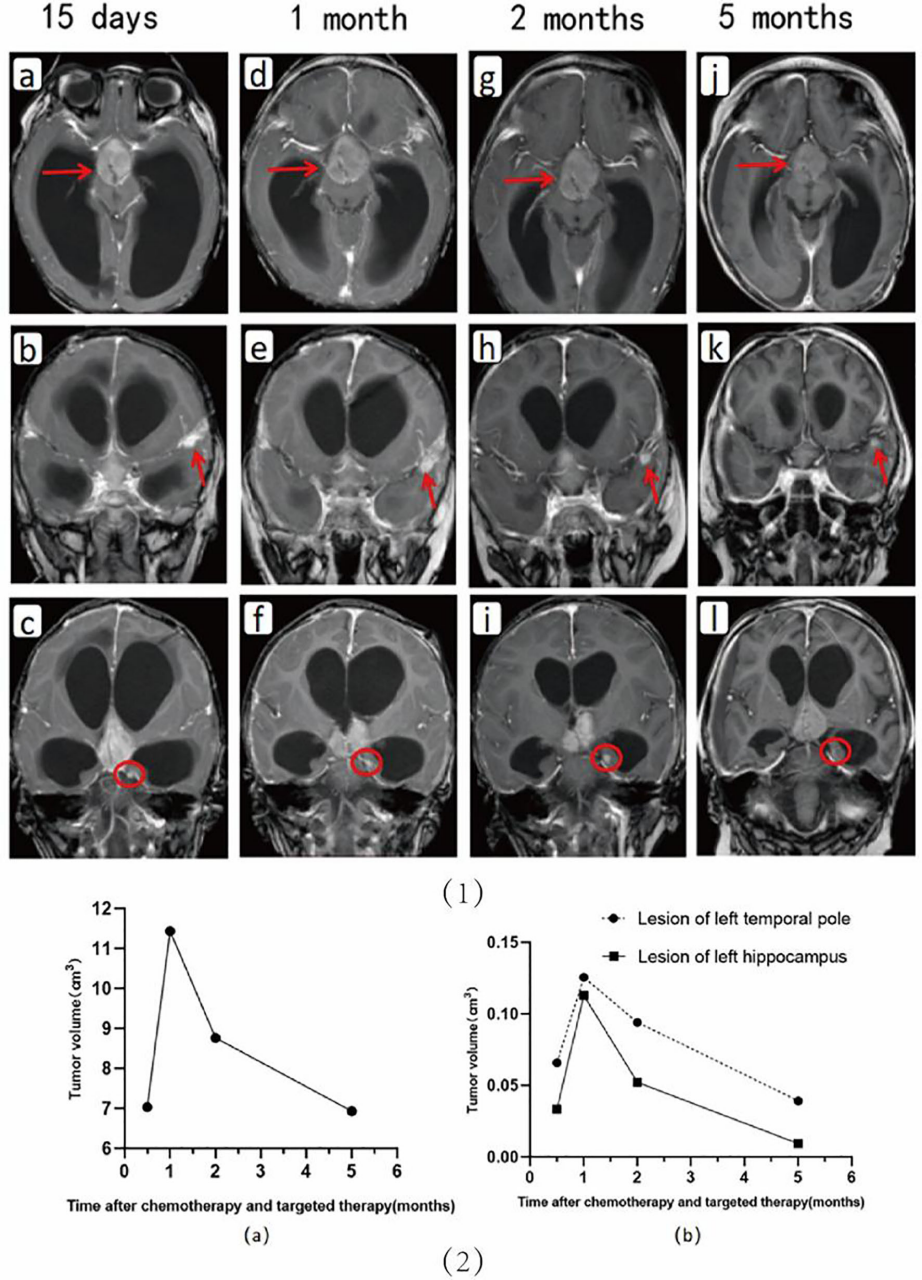
Upper: Changes in the cerebral lesions are seen on imaging during chemotherapy and anti-ALK therapy. Shown are images at 15 days **(A–C)**, 1 month **(D–F)**, 2 months **(G–I),** and 5 months **(J–L)** after the start of drug therapy, respectively. Lower: Shown are the volumetric changes in the supra-sellar lesion **(A)** and in the lesions in the left temporal pole and hippocampus **(B)** during chemotherapy and targeted therapy.

**Figure 3:**
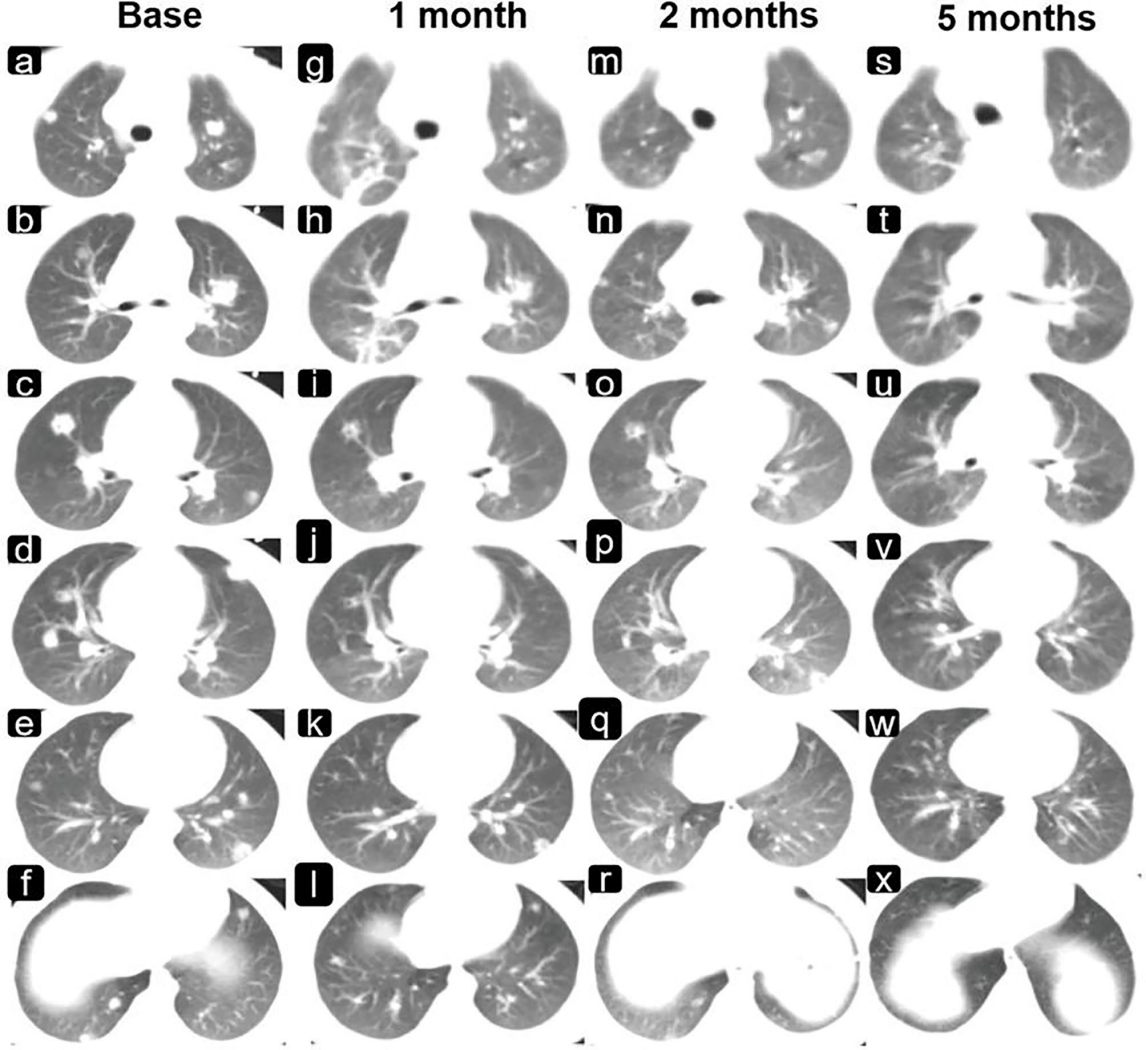
Changes in the lung lesions are observed on imaging during chemotherapy and anti-ALK therapy. Shown are images of the lungs **(A–F)** before drug therapy and **(G–X)** after drug therapy (**G–L**, 1 month; **M–R**, 2 months; and **S–X**, 5 months).

The changes in the intracranial lesions observed on MRI (PHILIPS ACHIEVA 1.5T) during chemotherapy and anti-ALK therapy are shown in **Figures 2 1A-L**. To quantify the changes in lesions, we calculated the volume as maximum height × maximum length × maximum width × π/6. The volume changes of the three lesions in the cerebrum are shown in **Figure 2-2A**. Although the tumors increased in size at the beginning of treatment, they gradually decreased with further treatment. The changes in the pulmonary lesions during treatment are shown in **Figures 2-2B**. As the treatment progressed, the tumor size in the lungs decreased significantly and seemed to show better efficacy than did the lesions in the cerebrum (**Figures 3G–X**).

### Histology

The histiocytes were mostly epithelioid, partly round or foamy, with abundant cytoplasm, and had a low nucleoplasm ratio. Proliferative lesions with lymphocytic infiltration and scattered glial cells were observed in the tissue cells. Immunohistochemical analysis confirmed the histiocytic nature of the lesional cells, which were positive for CD68/PGM-1, CD20 (small subset), CD3 (subset), CD163, CD30, ALK-1, CD4, and Cyclin D1 and negative for S100, CD1a, p53, CD34, CD99, TdT, CXCL13, EGFR, SSTR2, SALL4, PLAP, and Langerin. The Ki-67 proliferation index was 5%. ALK1 expression was positive, and next-generation sequencing analysis revealed a KIF5B (exon 24)-ALK (exon 20) fusion. Finally, the patient was diagnosed with ALK-positive histiocytosis.

### Treatment

Subsequently, the child underwent partial resection of the suprasellar lesion to obtain a pathological biopsy. After surgery, the child developed hydrocephalus and intracranial infection and subsequently underwent right lateral ventricular borehole drainage and Omaya capsule placement, along with aggressive anti-infective therapy (ceftriaxone 400mg qd) and levetiracetam (100mg bid ivgtt) for seizure control. After 46 days, the child’s condition stabilized, and he began to receive crizotinib (250mg qd po) for anti-ALK therapy and CHOP regimen (cyclophosphamide, doxorubicin, vincristine, prednisone) for chemotherapy. The patient received vincristine (0.35mg qiw ivgtt) and prednisone for induction chemotherapy for about 3 months. Subsequently, he received doxorubicin (15mg tiw ivgtt), vincristine (0.45-0.55mg qiw ivgtt), cyclophosphamide (100mg qd ivgtt) and prednisone for maintenance chemotherapy.

Intracranial infection of the child was progressively worsening. Cerebrospinal fluid culture was suggestive of *Bacillus cereus* 3 months later after the first surgery. Although the boy had received aggressive anti-infective treatment, including ceftriaxone, meropenem, and vancomycin combined with rifampin, along with external drainage of cerebrospinal fluid, the intracranial infection did not resolve completely. Unfortunately, the child died 8 months after the first surgery because of worsening intracranial infection.

## Discussion

Histiocytoses are characterized by inflammation and accumulation derived from the monocyte and macrophage lineages, causing damage to various tissues (8). In recent years, ALK-positive histiocytosis, a rare type of histiocytosis, has occasionally been reported. Since Chan et al. reported the first case in 2008, almost 20 cases have been reported to date. Herein, we report a case of ALK-positive histiocytosis invading the respiratory and central nervous systems and report the results of dynamic and detailed follow-up of tumor changes. Despite poor prognosis of the child, the case is informative.

Although the prognosis of ALK-positive histiocytosis is generally good according to the literature, the child in this study died of intracranial infection despite receiving surgery, chemotherapy, anti-ALK, and anti-infective therapy. **Table 1** summarizes the literature reports of ALK-positive histiocytosis invading the CNS, and most patients benefited from regular therapy. Chang et al. reported a case of ALK-positive histiocytosis invading the CNS, intestine, and bone marrow (3). Although the boy received steroids and chemotherapy, he died of systemic disease. Usually, ALK-positive histiocytosis is a benign disease that can be controlled with regular therapy, including surgery, chemotherapy, and anti-ALK therapy. However, it is vital to be aware of possible complications during therapy, which could be a major cause of treatment failure.

**Table 1:** Literature search results on the clinical features and outcomes of ALK-positive histiocytosis involving the CNS.

Citation	Age	Sex	Location in CNS	Site of involvement	Therapy	Follow-up/prognosis
Lucas et al. (6)	7 y	Female	Cerebellar vermis	No	Surgery	12 mo/well
Lucas et al. (6)	10 y	Female	Pericentral	No	Surgery	6 mo/well
Qiu et al. (1)	49 y	Male	Left temporal lobe, right frontal lobe	lungs, liver, bone pancreas, prostate, parotid gland, abdominal wall	GKRS, chemotherapy (lenalidomide), anti-ALK	2 mo/well
Rossi et al. (7)	10 mo	Male	Left frontal lobe, left parietal lobe	No	Surgery, chemotherapy (vinblastine), anti-ALK (alectinib)	7 mo/well
Rossi et al. (7)	11 y	Female	Right frontal lobe	No	Surgery	4 mo/well
Chang et al. (3)	33 mo	Male	NA	intestine, bone marrow	Steroids, chemotherapy (etoposide, cyclosporine, cytarabine, methotrexate)	2 mo/died
Chang et al. (3)	15 y	Male	Cavernous sinus	No	anti-ALK (crizotinib)	NA/well
Tian et al. (5)	51 y	Female	Left frontal lobe	Lungs, lymph nodes	Surgery, anti-ALK (alectinib)	10 mo/well
Takeyasu (4)	17 y	Female	Right frontal lobe	breast	anti-ALK (alectinib)	30 mo/well
Jaber (9)	27 y	Male	intradural extramedullary at the L3 level	No	Surgery	9 mo/well

ALK, anaplastic lymphoma kinase; GKRS, gamma knife radiosurgery; mo, months; y, years; NA, not available.

AS ALK-positive histiocytosis can spread throughout the multiple systems, it can also disseminate intracranially. Qui et al. reported a Caucasian man with ALK-positive histiocytosis (1). The lesions of the patient were located in the left medial temporal lobe, right inferior frontal lobe and dural-based parasagittal right parietal convexity (1). Rossi et al. also reported a child with the same disease whose lesions were located in frontal and parietal lobes (7). It appears that disseminated growth is a characteristic of ALK-positive histiocytosis.

In the present case, it was necessary to differentiate ALK-positive histiocytosis from other types of histiocytosis. Classic Rosai–Dorfman disease (RDD) presents with massive bilateral, painless cervical lymphadenopathy with associated fever, weight loss, and night sweats (10). The average age at onset of RDD is approximately 20 years. Vaidya et al. has summarized 19 patients with RDD and find that lymphadenopathy is a common feature in imaging and bilateral uniformly enhancing cervical lymph node enlargement is the predominant abnormality on FDG-PET scans (11). Histologically, enlargement of the node with extensive sinusoidal expansion visible under low magnification is characteristic of RDD (12). Cells with abundant wispy cytoplasm and emperipolesis can be seen, alone or mixed with inflammatory cells, including lymphocytes and plasma cells (10). In general, RDD presents with S100 protein (+) and ALK (-) on immunohistochemistry. However, it may arise in cases of ALK-positive histiocytosis with diffuse cytoplasmic positivity of S-100 protein, especially when emperipolesis is also present (13).

Erdheim–Chester disease (ECD) was first described in 1930, and 1500 cases have been reported (14). ECD usually occurs in adults, and patients are more likely to be women (15, 16). The neuroimaging presentation of ECD is diverse, with varying degrees of meningeal, cranial, cerebral, and spinal cord involvement (17). ECD is characterized by patchy intensification within the lesion, which can last for several days (17). The histology characteristic of ECD is multisystemic proliferation of mature histiocytes on a background of inflammatory stroma (18). Pathology may present loose clusters of classical foamy or granular histiocytes with well-defined cell borders (18). Fibrosis is present in most cases with a few Touton-type cells (18).

ALK-positive histiocytosis can occur in both children and adults, and may involve multiple systems. In the current study, enhancement at T1-weighted with intracranial dissemination is characteristic of the ALK-positive histiocytosis. Histologically, minimal cytologic atypia and focal spindled histiocytes are noted, along with scattered small lymphocytes and eosinophils (1). Touton-type multinucleated giant cells have been found in ALK-positive histiocytosis (9).

This case provides a new direction for the differential diagnosis of suprasellar tumors in radiology. In children with giant suprasellar tumors, the first diagnosis that comes to mind is craniopharyngioma (CP), which has two histological subtypes: adamantinomatous and papillary (19). The solid parts of CPs as well as calcified tissue and cyst walls can show a variety of T1 signals, from hypointense to hyperintense (20). CPs have generally been hypointense and hyperintense on T2­weighted images. In addition, germinoma is a common intracranial tumor of children, in which the AFP and β-hCG levels are generally significantly high. Germinoma shows low to equal signal on T1­weighted images and high signal on T2­weighted images, which could be associated with necrosis and cystic degeneration, and significant enhancement on contrast-enhanced T1-weighted MRI. The ALK-positive histiocytosis reported in this study had a solid component without calcification or cystic changes, and enhancement scans showed significant enhancement, which differed somewhat from the above two tumor types.

The efficacy of chemotherapy and anti-ALK therapy here appeared to be greater in the lung than in the cerebrum. During the limited follow-up period of the current study, the regression speed of lesions located in the lungs was faster than that of lesions located in the CNS. Tian et al. reported a middle-aged female with ALK-positive histiocytosis located in the left frontal lobe, lungs, and lymph nodes (5). The cerebral lesion was removed surgically, and the patient received anti-ALK therapy (alectinib). Lesions of the lungs showed gradual regression during treatment as well. The lesions of the patient from Qiu et al.’s study involved multiple systems including the brain, lungs, liver, prostate, and bone (1). He received gamma knife treatment, lenalidomide-based chemotherapy, pembrolizumab, and anti-ALK therapy. The lesion was reported as stable, but additional information about the follow-up was lacking. Crizotinib and alectinib is an ALK-tyrosine kinase inhibitor that has been applied in the treatment of non-small-cell lung cancer (21). Several patients with ALK-positive histiocytosis have been benefit from crizotinib and alectinib as well (3–5, 7). The presence of the blood–brain barrier may affect the action of ALK-tyrosine kinase inhibitor in the CNS, but there is insufficient evidence to support the hypothesis that the efficacy of chemotherapy and anti-ALK therapy is greater for ALK-positive histiocytosis in the lung than in the cerebrum. Further studies are needed to address this question.

We conclude that ALK-positive histiocytosis can involve the central nervous system and disseminate intracranially. It is also worthy of consideration for the differential diagnosis of suprasellar lesions. Once the diagnosis of ALK-positive histiocytosis is confirmed, chemotherapy and anti-ALK therapy should be considered, but patients should be alerted to a risk of fatal complications.

## Ethics Statement

We have obtained written informed consent from the patients’ legal representatives for the publication.

## Author Contributions

YG and YL proposed the conception and design of the study. H-Bq and GN conducted data collection. YG and F-lJ conducted the literature search and data extraction. YG, HL, and X-mM were involved in picture production. YG and YL drafted the manuscript. All authors contributed to the article and approved the submitted version.

## Conflict of Interest

The authors declare that the research was conducted in the absence of any commercial or financial relationships that could be construed as a potential conflict of interest.

## Publisher’s Note

All claims expressed in this article are solely those of the authors and do not necessarily represent those of their affiliated organizations, or those of the publisher, the editors and the reviewers. Any product that may be evaluated in this article, or claim that may be made by its manufacturer, is not guaranteed or endorsed by the publisher.
