# Supplementary material for: Case Report: ALK-Positive Histiocytosis With KIF5B-ALK Fusion in Cerebrum-Disseminated Lesions in a Child
Source: Front Oncol. 2022 Mar 10;12:858939. doi: 10.3389/fonc.2022.858939 (PMC8960947; doi:10.3389/fonc.2022.858939)
Supplement: Supplementary file 1 [file DataSheet_1.docx]

**Figure 1.** The timeline of the patient during treatment


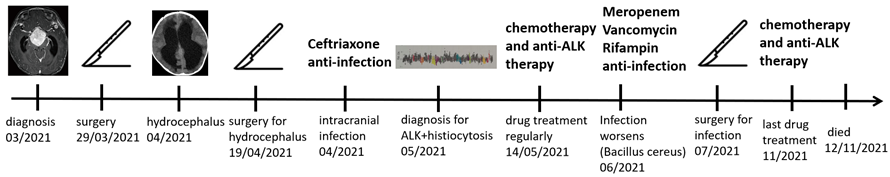


**Figure 2 (A, B)** Shown is a specimen from a patient with Rosai–Dorfman disease. **(A)** A low-power view of a node shows alternating light and dark areas. The dark areas comprise an admixture of lymphocytes and plasma cells. The light areas comprise the pale pink histiocytic cells characteristic of this disease (40×)^[9]^. **(B)** The node shows characteristic cells with abundant wispy cytoplasm and admixed inflammatory cells including lymphocytes and plasma cells. Emperipolesis is seen (200×) ^[9]^. **(C, D)** Shown are biopsies from a patient with Erdheim–Chester disease. **(C)** Loose clusters of classical foamy or granular histiocytes with well-defined cell borders are seen (200×). **(D)** Fibrosis can be present in most cases with few Touton-type cells (40×). **(E, F)** Shown is a specimen from a patient with ALK-positive histiocytosis. **(E)** Minimal cytologic atypia and focal spindled histiocytes are noted, along with scattered small lymphocytes and eosinophils (200×) ^[1]^. **(F)** Touton-type multinucleated giant cells are found in ALK-positive histiocytosis (200×)^[16]^. All sections are stained with hematoxylin and eosin.


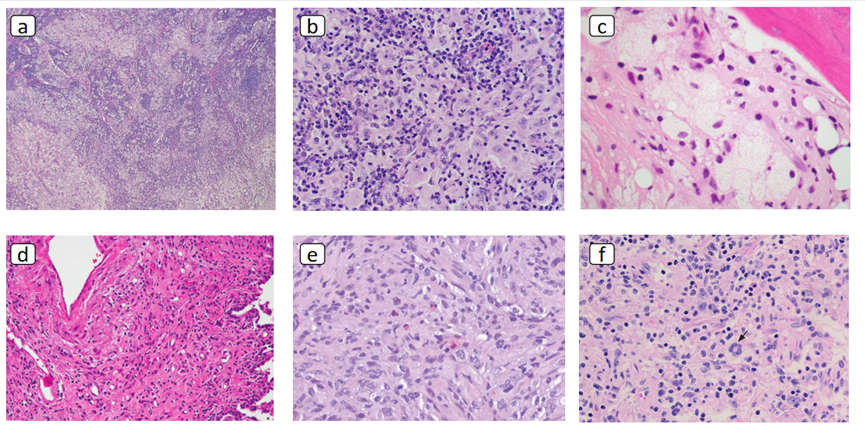


The parameters of the sequences in T1-weighted, T2-weighted, FLAIR, contrast-enhanced T1-weighted and DWI are shown as follows:

MRI before surgery: PHILIPS INGENIA 3.0T.

Sequences T1: TR= 2000 ms，TE= 20 ms, voxel size = 0.78×1.07×5 mm^3^.

Sequences T2: TR= 3670 ms，TE= 110 ms, voxel size = 0.7×0.7×5 mm^3^.

Sequences FLAIR: TR= 7000 ms，TE= 130 ms, voxel size = 0.85×1×5 mm^3^. Sequences contrast-enhanced T1: TR= 260 ms, TE= 4.6 ms, voxel size = 0.65×0.8×5 mm^3^.

MRI after surgery: Achieva 1.5T.

Sequences T1: TR= 550 ms, TE= 15 ms, Flip angle = 69°, slice thickness = 6mm, FOV = 203 mm×203 mm.

Sequences T2: TR= 5000 ms，TE= 120 ms, Flip angle = 90°, slice thickness = 6mm, FOV = 189 mm×189 mm.

Sequences FLAIR: TR= 6800 ms，TE= 120 ms, Flip angle = 90°, slice thickness = 6mm, FOV = 220 mm×220 mm.

Sequences DWI: TR= 2531.2 ms，TE= 88.5 ms, Flip angle = 90°, slice thickness = 6mm, FOV = 208 mm×208 mm.

Sequences contrast-enhanced T1: TR= 157.7 ms，TE= 2.5 ms, Flip angle = 80°, slice thickness = 6mm, FOV = 230 mm×230 mm
